# Sex‐specific effects of leptin administration to pregnant mice on the placentae and the metabolic phenotypes of offspring

**DOI:** 10.1002/2211-5463.12757

**Published:** 2019-11-25

**Authors:** Elena I. Denisova, Valeria V. Kozhevnikova, Nadezhda M. Bazhan, Elena N. Makarova

**Affiliations:** ^1^ Laboratory of Physiological Genetics Institute of Cytology and Genetics the Siberian Branch of the Russian Academy of Sciences Novosibirsk Russia; ^2^ Department of Physiology Novosibirsk State University Novosibirsk Russia

**Keywords:** developmental programming, leptin, mice, placenta, pregnancy

## Abstract

Obesity during pregnancy has been shown to increase the risk of metabolic diseases in the offspring. However, the factors within the maternal milieu which affect offspring phenotypes and the underlying mechanisms remain unknown. The adipocyte hormone leptin plays a key role in regulating energy homeostasis and is known to participate in sex‐specific developmental programming. To examine the action of leptin on fetal growth, placental gene expression and postnatal offspring metabolism, we injected C57BL mice with leptin or saline on gestational day 12 and then measured body weights (BWs) of offspring fed on a standard or obesogenic diet, as well as mRNA expression levels of insulin‐like growth factors and glucose and amino acid transporters. Male and female offspring born to leptin‐treated mothers exhibited growth retardation before and a growth surge after weaning. Mature male offspring, but not female offspring, exhibited increased BWs on a standard diet. Leptin administration prevented the development of hyperglycaemia in the obese offspring of both sexes. The placentas of the male and female foetuses differed in size and gene expression, and leptin injection decreased the fetal weights of both sexes, the placental weights of the male foetuses and placental gene expression of the GLUT1 glucose transporter in female foetuses. The data suggest that mid‐pregnancy is an ontogenetic window for the sex‐specific programming effects of leptin, and these effects may be exerted via fetal sex‐specific placental responses to leptin administration.

## Graphical abstract

Leptin administration to pregnant mice transiently inhibited growth rate in the foetuses of both sexes, decreased placental GLUT1 mRNA level only in female foetuses, and decreased placental weight and increased growth rate after weaning only in male offspring. Sex‐specific programming effect of leptin may be related to its different action on the placentas of male and female foetuses.

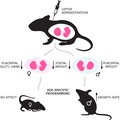

**Abbreviations**
BW: body weight
DIO: diet‐induced obesity
FI: food intake
GD: gestational day
GLUT1, GLUT 3: glucose transporters 1 and 3
IGF1, IGF2: insulin‐like growth factors 1 and 2
IGF2R: IGF2 receptor
SD: standard diet
*Slc2a1,Slc2a3*: genes encoding GLUT1 and GLUT3
*Slc38a1, Slc38a2, Slc38a4*: genes encoding SNAT1, SNAT2 and SNAT4
SNAT1, SNAT2, SNAT4: sodium‐coupled neutral amino acid transporters 1, 2 and 4

According to the ‘Developmental Origins of Health and Disease’ paradigm, the health of an individual depends on the intrauterine and early postnatal conditions 1. Obesity during pregnancy has been shown to increase the risk of metabolic diseases in the offspring 2. However, it remains unknown what factors of the maternal milieu induce alterations in the offspring phenotypes and the mechanisms that mediate the long‐term effects of these factors.

The adipocyte hormone leptin plays a key role in regulating energy homeostasis 3, and the circulating leptin levels are proportional to the adipose mass of the body 4. A considerable number of studies have demonstrated that leptin participates in developmental programming 5, 6, 7, 8. A protective effect against obesity in offspring was detected when leptin was administered throughout pregnancy or during the second half of pregnancy in rats and mice on a standard diet 5, 7, 9, 10. However, leptin administration during early pregnancy and mid‐pregnancy in food‐restricted mice was shown to increase the propensity of female offspring to develop diet‐induced obesity (DIO) 11. The programming effects of leptin probably depend on the timing of its administration, maternal nutrient conditions and the sex of the offspring.

Leptin may affect fetal development via its influence on placental functions 12. The placenta supplies foetuses with nutrients and growth factors, thereby determining the fetal growth rate and the offspring birth weight 13, 14. In turn, the birth weight predicts the future health of an individual. In humans, birth weights that are either too low or too high are associated with an increased risk of type 2 diabetes and obesity 15, 16. In mice, sexual dimorphism has been observed for the placental response to obesity during pregnancy 17, 18. This suggests that some obesity‐associated maternal factors (including elevated leptin) may differently affect the functions of the placentas of male and female foetuses. It is not known whether maternal leptin affects the fetal growth rate and the expression of nutrient transporters and growth factors in the placenta in mid‐pregnancy and whether the placental response to leptin action is sex‐specific.

The goal of the present study was to investigate the effects of leptin administration to mice during mid‐pregnancy on the offspring metabolic phenotypes, fetal weights and expression of genes in the placentas according to the offspring sex. We found that leptin administration to pregnant mice differentially affected some phenotypic traits of the male and female offspring, and this effect was associated with a temporary fetal growth restriction and a sex‐specific placental response to the leptin injections. We propose that the sex‐specific programming effect of leptin administered in mid‐pregnancy may be related to its different actions on the placentas of male and female foetuses.

## Methods

### Ethical approval

All experiments were performed according to Guide for the Care and Use of Laboratory Animals (1996) and the Russian national instructions for the care and use of laboratory animals. The protocols were approved by the Independent Ethics Committee of the Institute of Cytology and Genetics (Siberian Division, Russian Academy of Sciences).

### Diets

The standard chow diet was supplied in pellet form and was purchased from Assortiment Agro (Moscow region, Turacovo, Russia). Obesogenic food included sweet butter biscuits, lard and sunflower seeds. The animals received obesogenic food in addition to standard chow. The animals received excess quantities of each food including the chow, such that their intake was *ad libitum*.

### Animals

C57BL/6J mice were purchased from the Jackson Laboratory (Bar Harbor, ME, USA) and then were bred in the vivarium of the Institute of Cytology and Genetics. The mice were housed under a 12:12‐h light–dark (7 h 30 min–19 h 30 min light) regime at an ambient temperature of 22 °C. The mice were provided *ad libitum* access to commercial mouse chow and water.

The females were mated with the males at 10–14 weeks of age and were housed individually from the day a copulatory plug was detected (gestational day 0, GD0). On GD12, the females received subcutaneous injections of recombinant murine leptin (4.0 µg·g^−1^ BW) dissolved in saline to a final concentration of 500 µg·mL^−1^ or were injected with saline (control) at 9 h 00 min. According to our previous research, leptin administered at this dose to mice in late pregnancy did not affect the offspring viability but this administration influenced the offspring phenotype 5. The leptin‐ and saline‐administered mice were divided into three experimental groups.

In one group, 14 leptin‐treated and 17 saline‐treated females were weighed before and 24 h after injection and were sacrificed by rapid decapitation. Samples of their trunk blood were collected, and their foetuses and placentas were weighed. The fetal liver samples were collected and snap‐frozen in liquid nitrogen for subsequent sex determination.

In the second group, nine leptin‐treated and nine saline‐treated mice were sacrificed 7 h after leptin injection, and the samples of every placenta and fetal liver were snap‐frozen in liquid nitrogen. When the sexes of the foetuses were detected, the placentas from six leptin‐treated and six saline‐treated dams were selected to examine the placental gene expression. The selected dams had seven to nine foetuses and two or more foetuses of both sexes. Lightest and heaviest placentas within a litter were excluded from the analysis of gene expression as placental size was shown to influence on expression of genes encoding the transporters of glucose and amino acids 19.

The third group was used to examine the influence of leptin administration on the offspring phenotypes. At birth, the pups born to nine leptin‐treated and seven saline‐treated females were weighed, and the litters that contained more than seven pups were reduced to seven pups. The pup body weights (BWs) on postpartum days 1, 7, 14, 21 and 28 were measured. On postpartum day 28, two males and two females from each litter were separated from their mothers and housed individually, and their BWs and food intake (FI) were measured once a week until the age of 16 weeks. During this period, all animals were fed a standard chow diet *ad libitum*. From the age of 16 weeks, one male and one female from each litter continued to receive a standard diet, and one male and one female began to receive biscuits, lard and sunflower seeds in addition to the standard chow. This mixture mimics the cafeteria diet and potentiates the rapid development of obesity in mice 5. Mouse BWs were measured once a week over the course of 8 weeks. The consumption of obesogenic food was not measured. At the end of the experiment, the animals were sacrificed by decapitation, samples of blood were collected, and the weights of the abdominal fat pads were measured.

### Materials

Murine recombinant leptin was purchased from PeproTech (Princeton, NJ, USA).

### Plasma assays

Concentrations of leptin were measured with commercial kits (leptin: R&D Systems, Minneapolis, MN, USA, intra‐assay: 3.8–4.3%; interassay: 3.8–5%). Plasma glucose concentrations were measured using a commercial kit (Fluitest GLU; Analyticon Biotechnologies, AG 35104, Lichtenfels, Germany).

### Relative quantitative real‐time PCR

Total RNA was isolated from the individual placenta samples with TRI Reagent (Ambion, Carlsbad, CA, USA) according to the manufacturer’s instructions. Samples of placental RNA from every dam were pooled according to sexes of the foetuses. The pooled RNA (two samples for every dam extracted from two to three placentas of male and female foetuses) was used as a template to synthesize the first‐strand cDNA with Moloney murine leukaemia virus (MMLV) reverse transcriptase (Promega, Madison, WI, USA) and oligo(dT) as a primer. Applied Biosystems TaqMan gene expression assays (Igf1, Mm0043956_m1; Igf2, Mm00439564_m1; Igf2r, Mm00439576_m1; Slc2a1, Mm00441480_m1; Slc2a3, Mm00441483_m1; Slc38a1, Mm00506391_m1; Slc38a2, Mm00628416_m1; Slc38a4, Mm00459056_m1; ObRb‐LepR, Mm00440181_m1) with β‐actin as an endogenous control [TaqMan endogenous controls with FAM dye label and MGB mouse β‐actin (ACTB)] (Life Technologies Corporation, Carlsbad, CA, USA) and TaqMan Gene Expression Master Mix were used for relative quantitative real‐time PCR (Life Technologies Corporation). Sequence amplification and fluorescence detection were performed with the Applied Biosystems ViiA 7 Real‐Time PCR System. All expression assays were tested for reaction with a negative control (samples prepared without reverse transcriptase), and no fluorescence signals were detected during this verification. Mean CT values for β‐actin were equal in all experimental groups. Reactions were performed in duplicate, and the results were averaged. Relative quantification was performed by the comparative CT method, where CT is the threshold cycle.

### Determining of fetal sex

The sexes of the foetuses were determined using genomic DNA PCR with the following primers: SX_F, 5′‐GATGATTTGAGTGGAAATGTGAGGTA‐3′; SX_R, 5′‐CTTATGTTTATAGGCATGCACCATGTA‐3′ 20. DNA was extracted from fetal liver with a salt solution according to Aljanabi and Martinez (21) 19. The sex was determined in approximately 85% of the foetuses.

### Statistical analysis

General linear model nested design ANOVA was used with the factor ‘mother’ nested in the factor ‘maternal treatment’ to analyse the influence of maternal treatment on fetal and placental weights and offspring BWs during the period of maternal care. Repeated measures ANOVA was used to compare the BW, FI and FI/BW after weaning according to the maternal treatment (saline or leptin) differently in male and female offspring on standard diet. As two males and two females from each litter were used for this analysis, means per litter were identified and then statistical analysis was performed with this data. To examine the maternal influence on the response to a diet, BW and BW gain were analysed by repeated measures ANOVA with the factors ‘maternal treatment’ and ‘diet’ differently in male and female offspring. Two‐way ANOVA was used to analyse fat percentage and blood glucose concentrations with the factors ‘maternal treatment’ and ‘diet’ differently in male and female offspring or with the factors ‘sex’ and ‘diet’ differently in offspring of saline‐ and leptin‐treated mothers, and to analyse the expression of genes with the factors ‘maternal treatment’ and ‘foetal sex’. In addition, multiple comparisons were performed with the *post hoc* Newman–Keuls test. The comparisons between single parameters were performed with a two‐tailed Student’s *t*‐test. Significance was determined as *P* < 0.05. The statistica 6 software package (StatSoft, TIBCO Software Inc., Palo Alto, CA, USA) was used for analysis. The results are presented as the means ± SEM from the indicated number of mice or mean values.

## Results

### Influence of leptin administration to dams on GD12 on the weights of dams, their foetuses and their placentas

**Figure 1:**
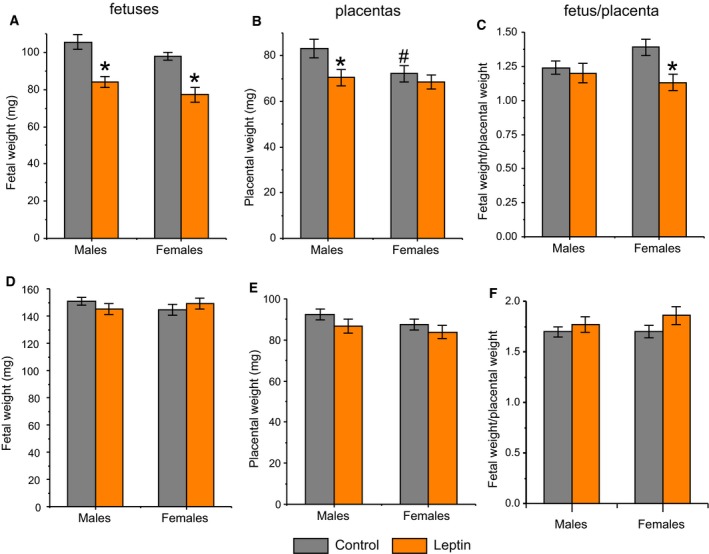
Influence of leptin administration to C57BL/6J mice on GD12 on the weight of foetuses and placentas within 7 h (A–C) and 24 h (D–F) after leptin injection. Data are presented as the means ± SEM from 18 male and 22 female foetuses of saline‐treated dams and from 33 male and 21 female foetuses of leptin‐treated dams within 7 h after leptin injection; from 55 male and 41 female foetuses of saline‐treated and from 38 male and 36 female foetuses of leptin‐treated dams within 24 h after injection. *P ≤ 0.05, maternal treatment, nested design ANOVA with the factor ‘mother’ nested in the factor ‘maternal treatment’; ^#^ *P* < 0.05, fetal sex in PBS‐treated dams, nested design ANOVA with the factor ‘mother’ nested in the factor ‘foetal sex’.

The effects of leptin on the weight of dams, their foetuses and placentas depended on the time that passed after the injection. Leptin‐ and saline‐treated dams did not differ in their BW changes (−1.2 ± 0.3%, *n* = 9; −1.1 ± 0.4%, *n* = 9; saline and leptin, respectively) but differed in the weight of their foetuses within 7 h after the injection. Compared to the results of the saline administration, the leptin administration resulted in a proportional reduction in the weights of the male foetuses (*P* < 0.001, nested design ANOVA) and their placentas (*P* < 0.01, nested design ANOVA); leptin administration also resulted in a reduction in the weights of the female foetuses (*P* < 0.05, nested design ANOVA) but did not affect the weights of the placentas of the female foetuses (Fig. 1A,B). As a result, the fetal/placental weight ratio became lower in the female foetuses of the leptin‐treated dams compared to that of the control dams (*P* = 0.05, nested design ANOVA, Fig. 1C). Within 24 h after injection, leptin administration reduced the dam’s BW gain by 2.35‐fold (Table 1), but the leptin‐ and saline‐treated dams did not differ in terms of their plasma leptin and glucose levels (Table 1). Nested design ANOVA did not reveal influence of the maternal treatments on either the fetal or placental weights within 24 h after the leptin or saline administration both in male and in female foetuses (Fig. 1D–F).

**Table 1:** Characteristics of the dams on GD13 within 24 h after the leptin or saline injection. The data are presented as the mean ± SEM for the indicated number of mice within the brackets.

	Saline	Leptin	*P* (Student’s *t*‐test)
Female BW gain (% of initial)	4.7 ± 0.7 (17)	2.0 ± 0.08 (14)	0.02
Litter size	6.8 ± 0.3 (17)	6.9 ± 0.5 (14)	NS
Plasma leptin concentrations (ng·mL^−1^)	6.3 ± 1.3 (8)	10.1 ± 2.2 (7)	NS
Plasma glucose concentrations (mm)	7.2 ± 0.4 (8)	7.5 ± 0.4 (7)	NS

### Influence of leptin administration to dams on GD12 on the offspring metabolic phenotypes

#### Offspring BW before weaning, and FI and BW after weaning

**Figure 2:**
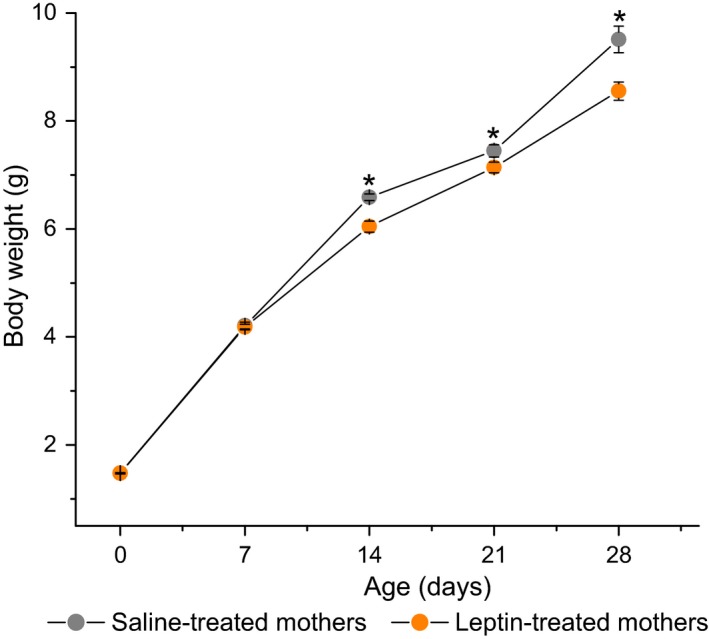
Influence of leptin administration to C57BL mice on GD12 on pup BW at the age of 1–28 days. Data are presented as the means ± SEM from 45 pups born to saline‐treated and from 60 pups born to leptin‐treated mothers. *P < 0.01, maternal treatment, nested design ANOVA with factor ‘mother’ nested in factor ‘maternal treatment’.

The administration of leptin to dams on GD12 did not affect fetal viability or weight at the end of pregnancy: the number and weights of newborn pups were equal in the leptin‐ and saline‐treated dams (Table 1, Fig. 2).

**Figure 3:**
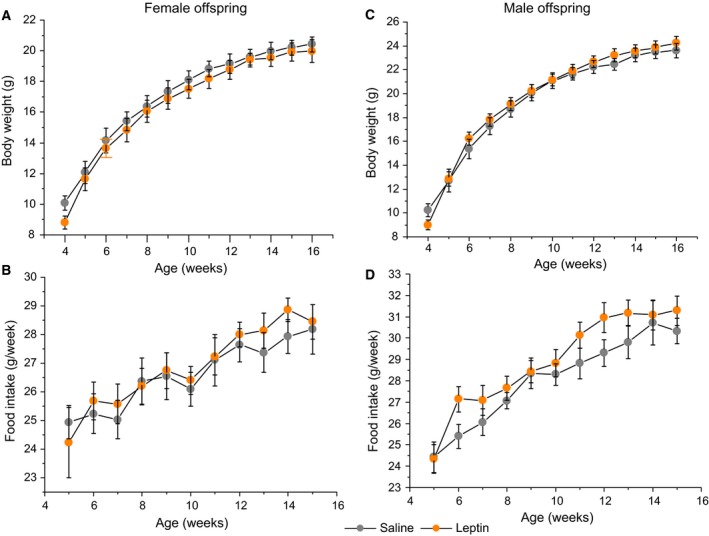
Influence of leptin administration to C57BL mice on GD12 on female (A) and male (C) offspring BW and female (B) and male (D) offspring FI after weaning. Data are presented as the means ± SEM from 7 values for offspring of saline‐treated mothers and 9 values for offspring of leptin‐treated mothers.

Leptin administration decreased the pup growth rate from postpartum days 7 to 28 and significantly decreased the BWs of both the female and male offspring before weaning (Figs 2 and 3).

After weaning, the offspring of the leptin‐treated dams demonstrated a growth surge that allowed them to catch up (Fig. 3). During the first 2 weeks after weaning, the male offspring, but not the female offspring, of the leptin‐treated dams had significantly higher weight gain than those of the control dams [5.66 ± 0.38 g (*n* = 12) and 7.31 ± 0.28 g (*n* = 17), *P* < 0.01, Student’s *t*‐test, for saline and leptin, respectively]. Leptin administration to pregnant dams did not affect the FI and BWs of the female offspring after weaning (Fig. 3A,B) but did increase the BWs of the male offspring after weaning (*P* < 0.01, repeated measures ANOVA, Fig. 3C). There were no significant differences in the FI (Fig. 3D) and FI:BW ratios for both the female and male offspring between the leptin‐treated and control mice (data not shown).

#### Offspring response to obesogenic diet

**Figure 4:**
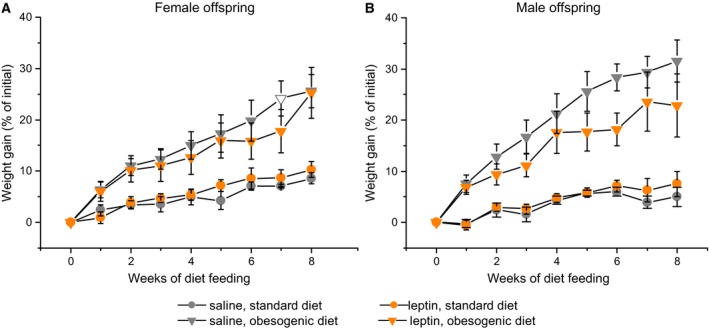
Influence of the obesogenic diet on BW changes in adult female (A) and male (B) offspring of mice that were administered leptin or saline on GD12. Data are presented as the means ± SEM from six to seven animals in every group.

**Figure 5:**
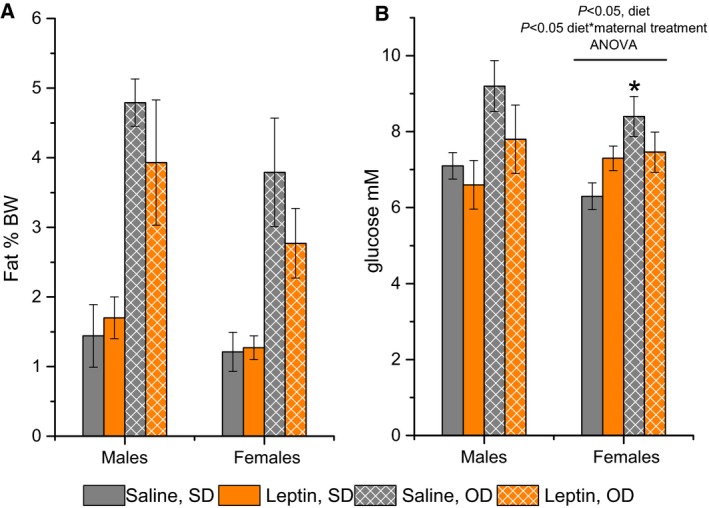
Influence of leptin administration to C57BL mice on GD12 on abdominal fat accumulation (A) and plasma glucose concentrations (B) in female and male offspring fed a standard (SD) or obesogenic (OD) diet. Fat accumulation was calculated as the ratio of abdominal fat weight to the BW and was expressed as %. Data are presented as the means ± SEM from six to seven animals in every group. Data were analysed by two‐way ANOVA with the factors ‘maternal treatment’ (saline or leptin) and ‘diet’ (standard or obesogenic) separately in males and females. The influence of diet and the interaction between diet and maternal treatment were detected for glucose concentrations in females with *P* < 0.05.*P < 0.05, SD vs. OD in females, *post hoc* Newman–Keuls test.

Obesogenic diet induced the development of obesity in both males and females (*P* < 0.001, factor ‘diet’, repeated measures ANOVA, Fig. 4A,B), and prenatal exposure to leptin did not significantly affect the BWs or BW gain of the female and male offspring. This leptin administration also did not affect abdominal fat accumulation in the male and female offspring regardless of diet (Fig. 5A) and abolished the presence of hyperglycaemia in obese animals. The blood glucose concentrations were higher in the DIO mice than those in the standard diet (SD) mice for the offspring of control mothers and were the same in the DIO and SD mice born to leptin‐treated mothers (two‐way ANOVA with factors ‘diet’ and ‘sex’, Fig. 5B). When glucose concentrations were analysed separately in males and females, the interaction effect of factors ‘maternal treatment’ and ‘diet’ was observed in females, but not in males.

### Influence of leptin administration on gene expression in placentas

**Figure 6:**
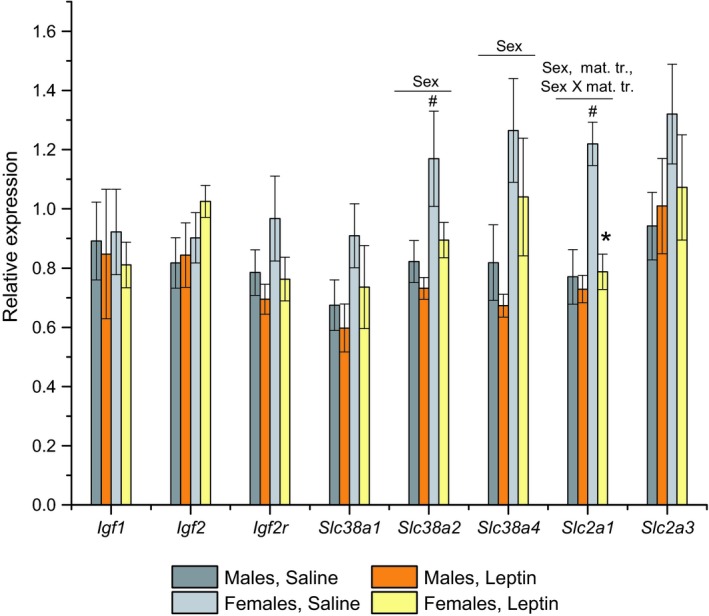
Influence of leptin administration to C57BL mice on GD12 on the relative gene expression levels in the placentas of male and female foetuses. Relative gene expression was estimated using reverse transcription and real‐time PCR analysis by the comparative CT method. Data are presented as the means ± SEM from six samples in every group. Data were analysed by two‐way ANOVA with the factors ‘maternal treatment’ (saline or leptin) and ‘foetal sex’, and the influence of fetal sex was detected for *Slc38a2*, *Slc38a4* and *Slc2a1* genes with *P* < 0.05. The influence of maternal treatment and the interaction between fetal sex and maternal treatment were detected for *Slc2a1* genes with *P* < 0.05. *P < 0.05, females, leptin vs. saline, ^#^ *P* < 0.05, saline, females vs. males, *post hoc* Newman–Keuls test.

The comparison of the gene expression in the placentas of male and female foetuses revealed sex‐dependent differences in the mRNA levels of the amino acid transporters SNAT2 (*P* < 0.05, *F*_1.20_ = 7.2, ANOVA) and SNAT4 (*P* < 0.05, *F*_1.20_ = 7.5, ANOVA) as well as sex‐ and treatment‐dependent differences in the mRNA levels of the glucose transporter GLUT1 (*P* < 0.01, *F*_1.19_ = 12.6 for sex; *P* < 0.01, *F*_1.19_ = 10.9 for maternal treatment; *P* < 0.05, *F*_1.19_ = 7.46 for sex X maternal treatment) (Fig. 6). In the control group, the relative expression levels of the *Slc38a2* (SNAT2) and *Slc2a1* (GLUT1) genes were significantly higher in the placentas of the female foetuses than the relative expression levels in the placentas of the male foetuses (Fig. 6), but these sex differences were not observed after leptin treatment. Leptin administration significantly decreased the placental mRNA levels of the glucose transporter GLUT 1 only in the placentas of the female foetuses.

## Discussion

In this study, we tested the assumption that the programming effect of leptin administered to mice during mid‐pregnancy may depend on the sex of the offspring. In addition, we suggested that any differences in the functional responses of the placentas of the male and female foetuses may underlie the sex‐dependent programming action of leptin. We injected leptin once on GD12 and evaluated both the acute response of the placentas and the foetuses to this injection and the long‐lasting effects of this administration on the metabolic phenotype of male and female offspring. Although a single injection of leptin does not imitate the leptin levels that are characteristic of obesity, this model allows us to assess whether the foetuses on day 12 of development are sensitive to the action of maternal leptin and to reveal an acute response from the foetuses and placentas. We found that a transient increase in the leptin levels in the blood of female mice at GD12 affects the offspring phenotype, and the effect on some phenotypic traits depended on the sex of the offspring.

We found that prenatal exposure to leptin during mid‐pregnancy did not prevent DIO development, but it did abolish hyperglycaemia in obese animals of both sexes. A previous study showed that hyperleptinaemia during pregnancy improved the glucose metabolisms of the offspring of DB/+ female mice 8. Our results are consistent with this finding and suggest that the specific period between gestational days 12 and 13 is critical for the development of glucose homeostasis systems in the mouse offspring. In mice, the peak of the proliferation of progenitors of hypothalamic leptin‐activated neurons that regulate energy balance and glucose homeostasis occurs on day 12 22, 23. Leptin has been shown to promote the migration and differentiation of hypothalamic neural progenitor cells 24. The leptin receptor Ob‐Rb was found in mouse fetal brain from postcoital day 10.5 25. These data suggest that increased leptin levels at the time of hypothalamic neuron birth may directly influence the formation of neuronal systems regulating energy balance and glucose homeostasis.

According to our previous results 5, the male offspring exhibited a higher sensitivity to prenatal leptin exposure than the sensitivity of the female offspring: the males born to leptin‐treated mothers became heavier than the control males at the age of 6 weeks due to an accelerated growth rate after weaning. However, leptin administration to female mice at the end of pregnancy had the opposite effect: it decreased both the food intake and BWs of the male offspring 5. Hyperleptinaemia throughout pregnancy also decreased the BWs of mouse offspring 5, 7. These findings suggest that the leptin programming action strongly depends on the stage of embryo development.

Although the programming effects of maternal leptin on the offspring postnatal phenotype has been well documented 5, 6, 7, 8, the effect of leptin on fetal growth and placental function was not studied well enough, and the role of leptin in the regulation of fetal growth remains unclear. The data obtained in humans and rodents indicate that leptin stimulates fetal growth: decreased leptin levels in food‐restricted pregnant rats are associated with fetal intrauterine growth restriction 26, 27, whereas elevated leptin levels in obese mice are associated with fetal overgrowth 28, 29, and *in vitro* leptin stimulates amino acid transport in human placentas at the end of pregnancy 30, 31. However, *in vivo* experiments on leptin‐treated pregnant mice and rats have shown that maternal leptin inhibits fetal growth 10, 32. Previously, we found that the weights of foetuses in mice with the A^y^ mutation (this mutation increases the blood leptin levels during pregnancy 33) were lower than those in the control mice on GD13 34. Our results are consistent with this previous finding and clearly demonstrate that maternal leptin inhibits fetal growth in mid‐pregnancy. We did not measure dam food intake in this study, but it seems unlikely that fetal growth inhibition resulted from leptin action on the food intake of pregnant dams. There was no association between changes in the weight of the foetuses and that of the pregnant dams in response to leptin administration; up until 7 h after the injection, the leptin‐ and saline‐treated dams did not differ in BW changes but differed in the weights of their foetuses, and through 24 h, the leptin‐treated dams gained less weight than saline‐treated dams did, but their foetuses did have a growth increase and caught up to the weights of the foetuses of control dams.

The effect of leptin administration on the growth of the foetuses is probably mediated via its influence on placental functions. Earlier in the genetic model, we found that an increased level of leptin in A^y^ mice was accompanied by a decrease in the weights of both the foetuses and the placentas 34.

In this study, we found that the placentas of male and female foetuses differed in size, gene expression and reaction to leptin administration. In control dams, the placental weight was significantly higher in the male foetuses than it was in the female foetuses. The same sex‐specific differences in placental weight were observed in mice on GD13 34 and GD18 35, 36, both in other species 37 and in humans 38. At the same time, the placental expression levels of genes encoding the glucose transporter *Slc2a1* (GLUT 1) and the amino acid transporters *Slc38a2* (SNAT2) and *Slc38a4* (SNAT4) were higher in the female foetuses than the expression levels in the male foetuses. These data coincide with the results of other authors who found an increased expression of the genes encoding GLUT1 and SNAT2 in small placentas compared with the expression in large placentas in mice during normal pregnancy 19. A decrease in the placental size in undernourished mice was associated with the increased placental expression of these genes 13. Our results suggest that the placentas of male and female foetuses that are different in size possibly adopt different strategies to support growth. According to this assumption, growth inhibition in male and female foetuses was associated with different placental responses to leptin administration as follows: the lowering of placenta weight in male foetuses and the inhibition of *Slc2a1* gene expression in placentas of female foetuses.

Interestingly, in A^y^ mice, elevated leptin levels were also associated with a decreased expression of *Slc2a1* in the placentas of only female foetuses 34. Taken together, our results indicate that the elevation of blood leptin levels in mice in mid‐pregnancy sex‐specifically inhibits the expression of gene encoding GLUT1 in placentas. GLUT1 is the most common glucose transporter in the placenta, and it regulates both the placental glucose uptake and the transplacental glucose transfer to the foetus 39. The influence of the leptin‐induced inhibition of *Slc2a1* expression on placental metabolism and transplacental glucose transfer may be the reason for growth retardation in female foetuses. Although leptin plays an important role in placental physiology 40, until now, there is no evidence that leptin influences the expression of the *Slc2a1* gene in the placenta. Leptin influence on the expression of the *Slc2a1* gene in placentas is possibly mediated by some metabolic signals that are generated by dams in response to leptin administration.

Leptin administration inhibited fetal growth without affecting the placental gene expression of *Igf1* and *Igf2*, which are known to affect fetal growth and placental morphogenesis, substrate transport and hormone secretion 41. The proportional decrease in the weight of both male foetuses and their placentas after leptin administration suggests that the reduction in physical size of the placentas can be a major cause of leptin‐induced growth retardation in male foetuses. The different effects of exogenous leptin on the weight of mouse placentas were shown depending on the stage of gestation and maternal diet: leptin decreased fetal and placental weight in mice on standard diet in late pregnancy 32 and increased placental weight in food‐restricted mice in early pregnancy 42. Our results agree with the observation of Yamashita *et  al*., although the mechanisms underlying the influence of the short‐term leptin increase in maternal blood on the placental weight are unclear.

In summary, leptin administration on GD12 has a long‐term effect on the metabolic phenotype of the offspring. Regardless of sex, this administration does not predispose the offspring to developing DIO, but it does prevent the adverse effects of obesity on blood glucose levels. These data confirm the role of leptin as a factor involved in developmental programming and demonstrate that the period between days 12 and 13 of embryonic development is critical for the formation of glucose homeostasis systems in mice. Male offspring exhibited a higher sensitivity to prenatal leptin exposure compared to the sensitivity of female offspring. The sex differences in the programming effect of leptin on offspring metabolic phenotypes may be related to the different responses of the placentas of male and female foetuses to leptin administration. Leptin administration transiently inhibited the growth rate in the foetuses of both sexes, and this effect was associated with a decreased placental weight in male foetuses and a decreased placental expression of the gene encoding the glucose transporter GLUT1 in female foetuses. This sex‐specific placental response to leptin administration may provide different developmental trajectories in male and female foetuses. Further investigation of the molecular pathways that link leptin‐induced changes in placenta with offspring development is necessary to understand the mechanisms underlying the sex‐specific programming effect of maternal leptin.

## Conflict of interest

The authors declare no conflict of interest.

## Author contributions

NMB and ENM conceived and designed the project; EID, VVK and ENM acquired the data; EID, VVK and ENM analysed and interpreted the data; and EID and ENM wrote the paper.
